# Hybrid membrane of flat silk cocoon and carboxymethyl chitosan formed through hot pressing promotes wound healing and repair in a rat model

**DOI:** 10.3389/fbioe.2022.1026876

**Published:** 2022-11-01

**Authors:** Baiqing Wu, Xiaoling Tong, Lan Cheng, Sha Jiang, Zhi Li, Zheng Li, Jiangbo Song, Fangyin Dai

**Affiliations:** ^1^ State Key Laboratory of Silkworm Genome Biology, College of Biotechnology, Southwest University, Chongqing, China; ^2^ College of Veterinary Medicine, Southwest University, Chongqing, China

**Keywords:** carboxymethyl chitosan, hot pressing, wound healing, rat model, flat silk cocoon

## Abstract

Clinical wound management is always a relatively urgent problem. Moreover, wounds, especially severe wounds with excessive tension or excessive movement are prone to tissue infection, necrosis, and other negative effects during healing. Therefore, research has aimed to develop low-cost complementary treatments to address the urgent need for an innovative low-cost dressing that can adapt to high mechanical requirements and complex wound conditions. At present, tissue engineering to produce artificial skin with a structure similar to that of normal skin is one effective method to solve this challenge in the regeneration and repair of serious wounds. The present study hot pressed flat silk cocoons (FSC) with carboxymethyl chitosan (CMCS) to generate a cross-linked binding without enzymes or cross-linking agents that simulated the 3D structural composites of the skin cuticle. This hybrid membrane showed potential to reduce inflammatory cells and promote neovascularization in skin wound repair. After hot pressing at 130°C and 20 Mpa, the FSC/CMCS composite material was denser than FSC, showed strong light transmission, and could be arbitrarily cut. Simulating the normal skin tissue structure, the hybrid membrane overcame the poor mechanical properties of traditional support materials. Moreover, the combination of protein and polysaccharide simulated the extracellular matrix, thus providing better biocompatibility. The results of this study also demonstrated the excellent mechanical properties of the FSC/CMCS composite support material, which also provided a low-cost and environmentally friendly process for making dressings. In addition, the results of this study preliminarily reveal the mechanism by which the scaffolds promoted the healing of full-thickness skin defects on the back of SD rats. *In vivo* experiments using a full-thickness skin defect model showed that the FSC/CMCS membranes significantly promoted the rate of wound healing and also showed good effects on blood vessel formation and reduced inflammatory reactions. This bionic support structure, with excellent repair efficacy on deep skin defect wounds, showed potential to further improve the available biomaterial systems, such as skin and other soft tissues.

## 1 Introduction

The skin is a coated barrier that protects the internal organs from harsh environments. Wounds account for 30% of the total emergency caseload in veterinary healthcare centers (Saito and Rhoads, 2003). Recent research in the medical and biomaterial fields has aimed to develop new scaffold materials for skin tissue engineering to allow the rapid healing of skin lesions and effective repair of the tissue functions of skin defects, especially skin defects that are difficult to cure. Regarding biological materials, the ideal artificial skin repair dressing should meet the following requirements: cells should easily adhere to the wound and the materials should be allowed to grow inside the wound. The material itself and the degradation products require controllable degradability. The repair dressing should not have cytotoxicity or cause a host immune response. The materials should be rich in resources, low in price, and provide biocompatibility without pathogenic viruses, bacterial infection, risks of disease transmission, and concerns regarding social ethics, etc. Skin-like three-dimensional scaffold structures should also have excellent mechanical strength to provide a framework and structural support for cells (Kundu et al., 2013; Xu et al., 2015).

**FIGURE 1:**
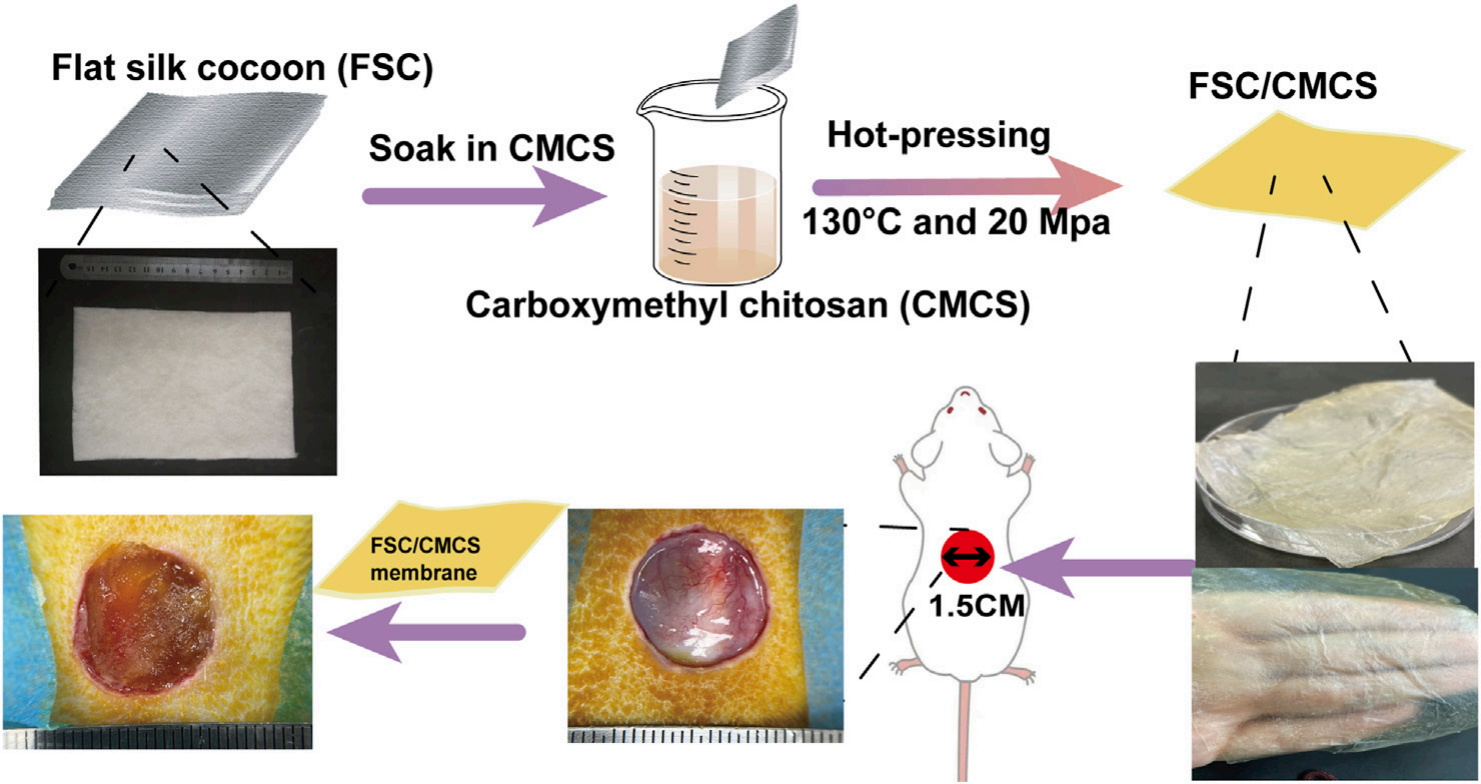
Illustration of the FSC/CMCS synthesis process and the use of FSC/CMCS as a wound dressing.

Silkworm cocoon is a natural polymer fiber composite made from single silk filaments and sericin binder (Alessio and Antonella, 2022). The structure and mechanical properties of the native silkworm cocoon have attracted research attention. The specific structure and excellent mechanical properties make the cocoon a good model for biomimetic engineering applications and a potential eco-friendly alternative reinforcement material for engineering composites. Silkworm cocoon shell (SCS) has been treated and used as the base substrate for dressing materials to promote wound healing. Silk fiber is widely used as a reinforcing material due to its low density, outstanding mechanical property, and sound sustainability to enhance the strength of composite materials (Yang et al., 2022). However, the direct use of silk cocoons as a wound dressing for large areas of damaged skin is challenging due to their small size and ellipsoidal shape. To address these issues, this study produced flat silk cocoons (FSC) by placing mature silkworm larvae on 2D spinning tools to produce novel natural silkworm cocoon membranes with controllable structure and size. Unlike common silk fibroin, FSC can be used directly, without requiring fibroin dissolution and regeneration. The advantages of FSC include a controllable size, excellent mechanical properties, and porous layer structure. Although few studies have reported on the use of FSC in the field as a biological material, particularly for the healing of wounds, it has demonstrated significant advantages as a composite matrix (Zhou et al., 2020) (Figure 1). In addition, silk (including sericin) can effectively promote the regeneration of wound tissues with its great biocompatibility and antibacterial and antioxidant properties. Thus, FSC has attracted increasing attention as a biomedical material for wound repair and regeneration (Chen et al., 2012; Agrawal et al., 2013; Wang et al., 2018a; Ling et al., 2018). The amino acids in silk sericin (SS) are similar in content and type to the natural moisturizing factors of the skin (sericin consists of 18 kinds of amino acids, including serine, glycine, and lysine, etc.) (Zhao et al., 2014). Sericin is a waste product in the silk degumming process, which is unfortunate because sericin is a biocompatible and biodegradable natural biopolymer that promotes adhesion and cell reproduction, and also shows antioxidant, hygroscopic, antimicrobial, and UV resistance properties, which can improve the production of extracellular matrix and promote wound healing (Kim et al., 2012; Ersel et al., 2016; Liang et al., 2017; Alessio et al., 2021a). Moreover, hot pressing treatment can improve the structure uniformity and solidity of FSC while maintaining the natural compound structure. The mechanical properties can also be greatly improved (Wang et al., 2017). Therefore, FSC have broad prospects in the field of artificial composite materials.

Carboxymethyl chitosan (CMCS) is the chitin derivative of chitosan formed by a methylation reaction (Chen and Park, 2003), to produce a water-soluble, non-toxic, antibacterial, and degradable compound. With numerous functional chemical groups, as a scaffold material for tissue regeneration, CMCS can provide mechanical support and promote cell attachment, proliferation, and differentiation. CMCS is a good biomedical material owing to its special characteristics, such as hemostatic effects, biodegradability, biocompatibility, and non-toxicity; thus, it has been used extensively in drug delivery systems, wound dressing materials, and tissue engineering scaffolds (Sakai et al., 2008; Bhardwaj and Kundu, 2011; Li et al., 2018; Lu et al., 2018).

Hot pressing is a simple and common method used to produce thermoplastic composites. During this process, the material undergoes deformation, matrix flow, and curing of the composite structure. Many studies have explored a variety of films and nanoscale architectures of silk fibroin and silk fiber. In addition, enzymatic or crosslinking agents have been used for material cross-linking as constructing larger, macroscale objects of fibroin and silk fiber has been challenging. However, these limitations could be overcome through the use of sintering and hot pressing methods (Alessio et al., 2019; Alessio et al., 2021b). Moreover, the preparation method is simple and environmentally friendly without requiring the use of organic solvents or cross-linking agents.

Recent studies on silk and CMCS as base materials in skin tissue engineering have demonstrated their feasibility as scaffold materials for repairing skin defects. The material properties can also be optimized by mixing or other special methods, thus taking full advantage of both materials. In preparing the composite materials, inspired by the unique structure of FSC, in which sericin can dissolve and vitrify under certain high temperatures (Wang et al., 2017), we developed a new and easy-to-implement technique for the direct preparation of FSC/CMCS wound dressings to meet the high mechanical requirements and complex wound conditions in animals. In this method, FSC were immersed in a CMCS solution to prepare an FSC/CMCS composite membrane *via* hot pressing. This material completely retained the dissolved sericin, rather than preparing the material indirectly through the regenerated silk fibroin membrane. The preparation method is simple and environmentally friendly, without requiring organic solvents or cross-linking agents. We synthesized FSC/CMCS composites under a heating temperature of 130°C and a pressure of 20 Mpa and systematically assessed their structures, mechanical properties, and biocompatibility. Compared to FSC alone (without CMCS), the tissue regeneration rate and wound closure rate were measured to evaluate the effects in promoting wound healing.

## 2. Materials and methods

### 2.1 Materials

FSC was provided by the *Bombyx mori* gene bank. Mouse L929 fibroblasts were provided by the stem cell bank of the Chinese Academy of Sciences (Shanghai). CMCS was provided by Shanghai Macklin Biochemical Co., Ltd.

### 2.2. Composite fabrication

The silkworm species were selected and fed until reaching the fifth instar. Approximately 5 kg/m^2^ of silkworms were confined on a two-dimensional plate after defecation for the spinning process. CMCS was dissolved in deionized water at 100 mg/ml. Then, based on the following method (Figure 1), FSC (thickness: 0.5 mm) was immersed in the CMCS solution and naturally dried. To soften the sericin and ensure the sound mechanical properties of the FSC, the test samples were moved to a plate vulcanizing machine for hot pressing at 130°C and 20 Mpa for 90 s (Wang et al., 2018b). The samples included FSC without CMCS (FCS-H) and FSC with CMCS (FSC/CMCS).

### 2.3 Structural and morphological characterization

#### 2.3.1 Morphological observation by scanning electron microscopy

The samples were first freeze-dried in a LYOQUEST-55 lyophilizer (Telstar, Terrassa, Spain). The dried samples then were fixed in the sample holder with a conducting resin and gold was applied to improve the electrical conductivity. A TM4000 desk scanning electron microscope (Hitachi, Tokyo, Japan) was used to observe the morphology of the samples under an accelerating voltage of 10 kV.

#### 2.3.2 Conformational structure analysis

To observe the surface morphology of the FSC and FSC/CMCS, each sample containing the same mass of 0.04 g FSC (25 × 15 mm) was scanned by electron microscopy. A Bruker alpha FT-IR spectrometer (Karlsruhe, Germany) was used to analyze the structural changes of FSC, FSC-H, FSC/CMCS, and CMCS (100 mg/ml CMCS solution was naturally dried and then transferred to a plate vulcanizer for 90 s after hot pressing at 130°C at 20 Mpa). The samples were ground and fully mixed with potassium bromide to make pellets. The infrared spectrum of the microspheres was obtained by scanning at 400–4,000 cm^−1^. To assess the secondary structure of the samples, peak-fitting software (Peakfit, v4.12) was used to fit the curve of the deconvolution spectrum of the samples with Gauss and Lorentz Amp functions. Finally, the content (%) of each secondary structure was calculated quantitatively (Alessio et al., 2021c).

The wide-angle X-ray diffraction (XRD) patterns of the dried sheets were recorded on an XRD diffractometer (X’Pert PRO XRD, PANalytical, Almelo, the Netherlands; Cu K-alpha radiation, 40 kV, 40 mA, *λ* = 0.15418 nm) in the two-theta angle range of 10°–80°. Thermogravimetric analyses of the freeze-dried samples (5 mg) were performed using a TG209 instrument (NETZSCH, Selb, Germany) under a nitrogen atmosphere at 40–800°C at a heating rate of 20°C min^−1^.

#### 2.3.3 Mechanical tensile testing

The tensile strengths of the FSC and series of FSC/CMCS were measured using an electromechanical universal testing machine (model: E 44.104, MTS systems (China) Co., Ltd.) The strip-shaped samples (45 × 20 × 0.1 mm^3^, length × width × thickness) were measured at a 5 mm/min stretching speed until rupture. The subsequent stress–strain curves were used to extract the tensile data of each sample.

### 2.4 *In vitro* cytotoxicity

Biocompatibility is an important premise of the application of biological materials. The CCK8 test is used to assess the proliferation and cytotoxicity of living cells. According to the extraction conditions in the ISO 10993-12, the sterilized freeze-dried SFH samples were placed in sterile six-well plates and immersed in sterile Dulbecco’s modified Eagle’s medium (DMEM) with fetal bovine serum (Gibco, Gaithersburg, MA, United States) in proportion (0.1 g ml^−1^). After incubation for 12 h and 48 h, cell viability was evaluated using a Cell Counting Kit-8 kit (Yeasen Biotechnology, Shanghai, China) according to the manufacturer’s protocol. The working principle of CCK8 is as follows: the reagent contains WST-8, a 2-(2-methoxy- 4-nitrophenyl)-3-(4-nitrophenyl)-5-(2,4-disulfonate benzene)-2H-tetrazolmonosodium salt, which is reduced by dehydrogenase in cells under the action of electron carrier 1-methoxy-5-methylphenolinium dimethyl sulfate to form yellow formazan with high water solubility. The amount of generated formazan is proportional to the number of living cells. Hence, this characteristic can be directly applied to the analysis of cell proliferation and toxicity. We tested this characteristic of FSC-H and FSC/CMCS membrane in L929 cells.

### 2.5 *In vivo* wound healing study

#### 2.5.1 Full-thickness skin wound model in rats

The Institutional Animal Care and Use Committee of Southwest University approved the study protocol. Healthy Sprague–Dawley mice (male, weight of 200–220 g) were selected for the construction of a full-thickness skin wound model. After anesthesia, the operation site (the skin of the back at the central spine) was cleaned with fluoride iodide. A round full-thickness wound 1.5 cm in diameter was then made according to the template line. The tissue to sarcolemma was taken and divided into three groups: the FSC-H-covering group, the FSC/CMCS-covering group, and the blank control group (covered with Vaseline cream). The dressings were fixed with sutures. The experimental animals were raised in single cages to avoid other animals licking the wounds. The SD mice were observed to inspect whether the wound materials fell off. All animal experiments conformed to the ethical regulations of the organization and were approved by the National Animal Science Experiment Teaching Center, College of Animal Science and Technology of Southwest University.

#### 2.5.2 Observation of the healing wound surfaces

The diets and activities of the rats were observed at 7 days, 14 days, and 21 days after the operation. Skin swelling or exudation at the suture or rejection reactions to the implanted scaffold materials were also inspected, along with wound healing. After sacrifice, the skin was cut along the original incision to observe the growth conditions of the materials and surrounding tissues, as well as the general healing of the wound. Objective evaluation was also performed. Wound images of all groups were obtained. The wound areas were calculated using ImageJ software. The wound healing rate (WH) was calculated according to the following formula in which S_0_ refers to the full-thickness skin damage area during the operation and S_1_ refers to the wound area at a time point:

$$WH\left(\%\right)=S_{0}-S_{1}/S_{0}\times 100.$$ (1)

#### 2.5.3 Histology and immunohistochemistry analyses

The granulation tissue sample was removed from neutral formaldehyde and then dehydrated and paraffin-embedded. According to the standard scheme, 5-mm serial sections were obtained using a microtome, before H&E and immunohistochemical staining. The immunohistochemical sections were stained with CD31 and TNF-α to observe the number of micro-vessels and positive areas (Xu et al., 2021). The H&E and immunohistochemical staining sections were observed on an optical microscope (BO-M30; AOSVI, Shenzhen, China).

### 2.6 Statistical analysis

Statistical analysis was performed using IBM SPSS Statistics for Windows, version 22.0. The data were analyzed statistically using Student’s t-tests. *p* < 0.05 was considered statistically significant. The data are expressed as means ± the standard error of the mean (SEM).

## 3 Results

### 3.1 Scanning electron micrography

**FIGURE 2:**
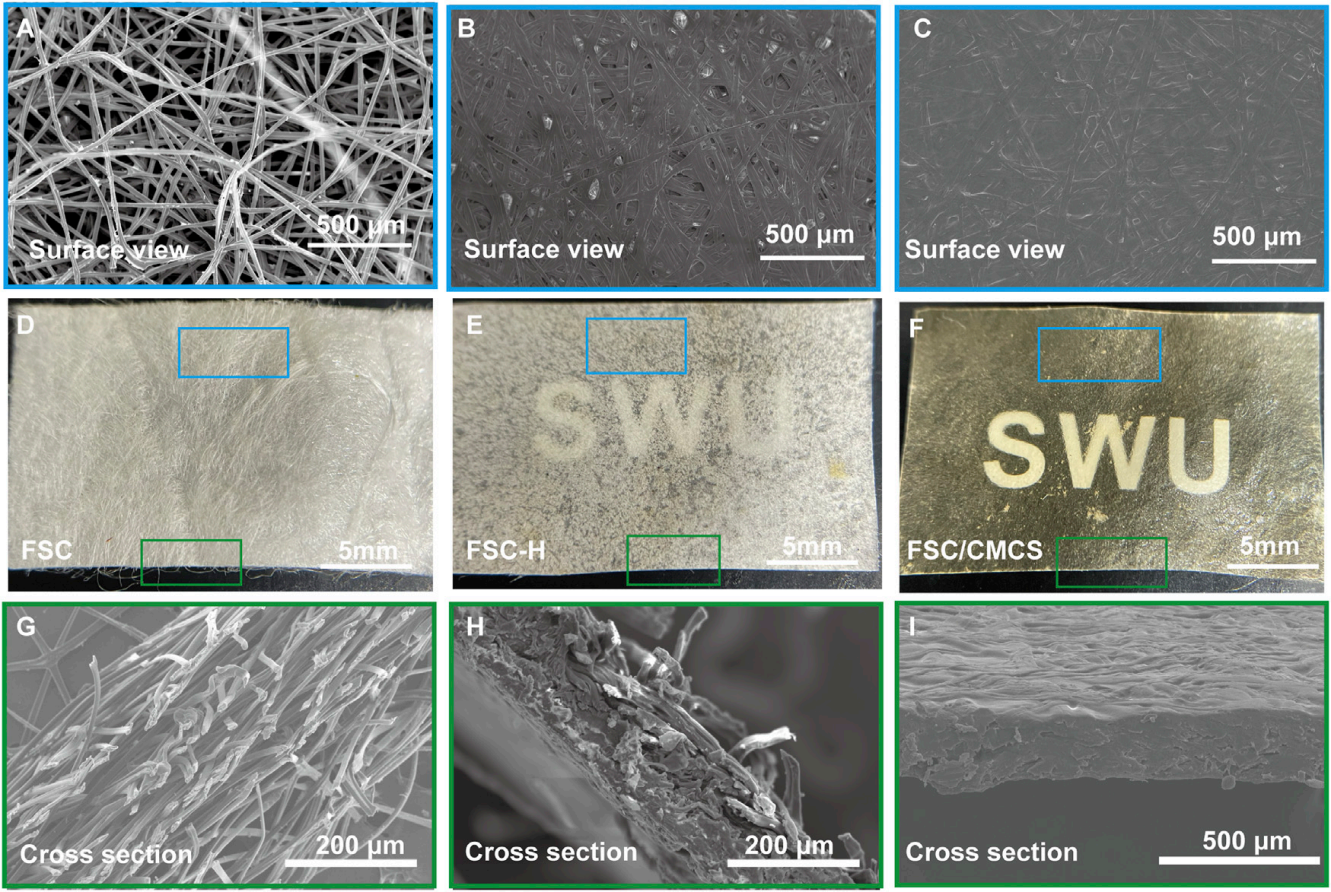
Digital and SEM images of the structures and morphologies. **(A–C)** Corresponding microstructure from the surface view. **(D–F)** Macroscopic presentations of FSC, FSC-H, and FSC/CMCS membranes. **(G–I)** Corresponding microstructure from a cross-sectional view.

FSC are similar to normal cocoons in structure, with a multilayered structure parallel to its surface (Figures 2A,D). The silk fibers of FSC remain independent in surface area and number of layers (Figure 2G). The multilayered structure of the silkworm cocoon is easily separated, mainly because there are fewer silk fibers in the connecting layer than those aligned in a single layer (Chen et al., 2012). Compared to FSC, the silk fibers at 130°C and 20 Mpa were slightly bent (Figures 2B,E), the thickness decreased obviously, the structure was denser, and the individual morphology of the fibers remained clear with many gaps remaining between the fibers. The gaps and single fibers were clearly observed on the FSC cross-section (Figure 2H), but the cracks were significantly reduced in FSC/CMCS. The surfaces of FSC-H and FSC/CMCM did not clearly show sericin fiber (Figures 2C,I).

### 3.2 Conformational structure analysis

#### 3.2.1 FT-IR characterization

**FIGURE 3:**
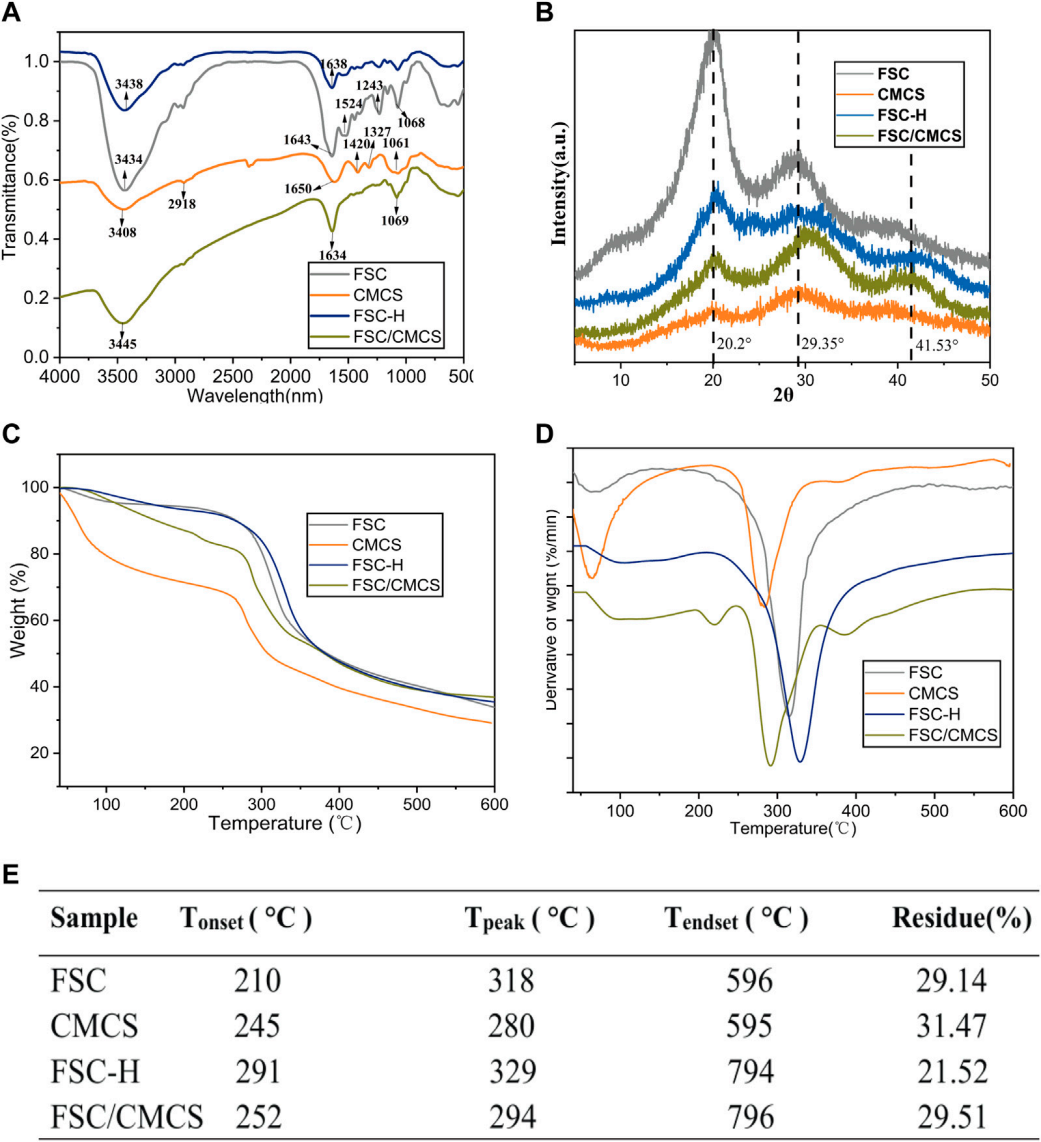
FSC and FSC/CMCS characterization. **(A)** Fourier-transform infrared spectroscopy spectra of FSC and FSC/CMCS. **(B)** X-ray diffraction pattern (XRD) of different membrane samples. **(C)** Thermogravimetric (TG) and **(D)** derivative thermogravimetric (DTG) of membrane samples. **(E)** summarized table of transition temperature and remaining. T_onset_: temperature at which thermal degradation begins; T_peak_: temperature at which thermal degradation peaks; T_endset_: temperature at which the process is complete.

**FIGURE 4:**
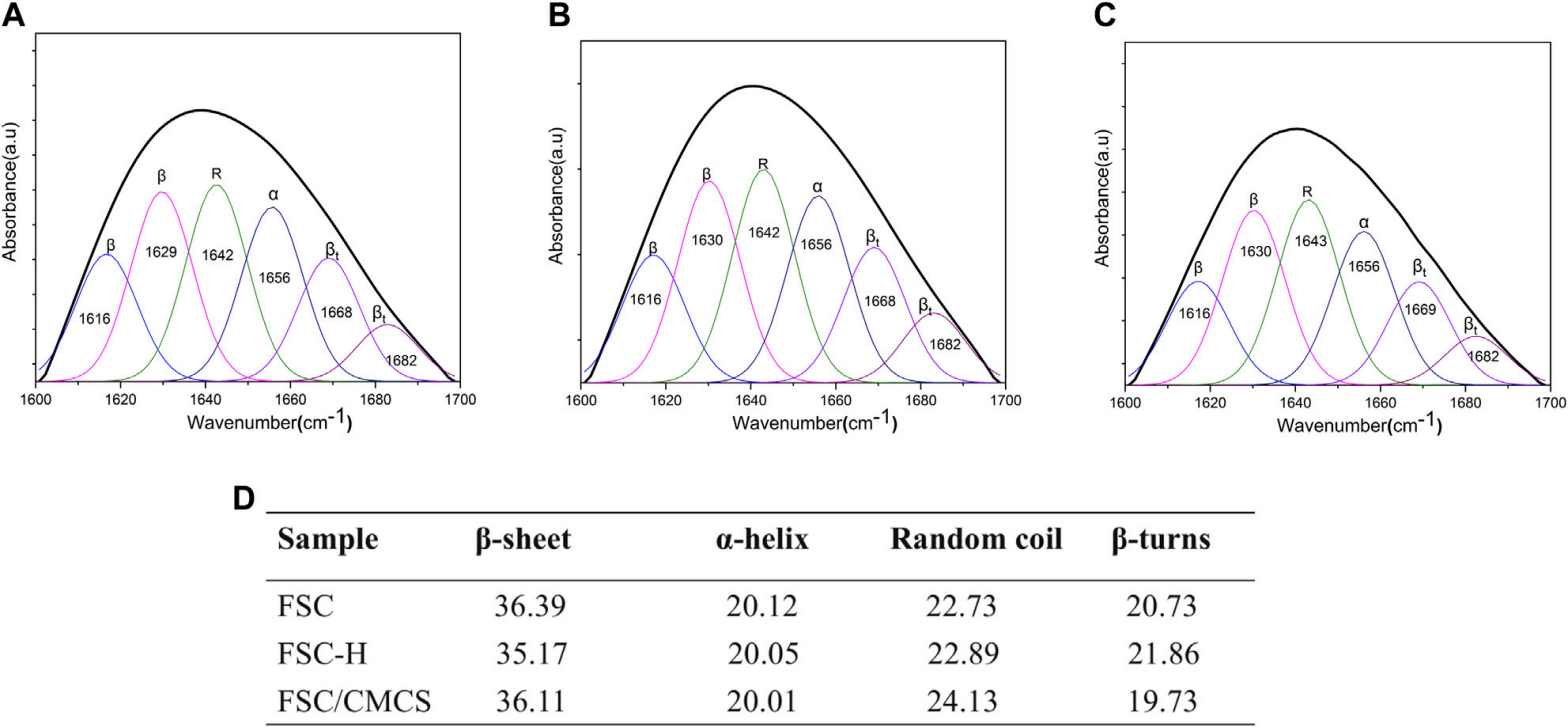
Quantitative analysis of the amide I bands in the samples. **(A)** FSC. **(B)** FSC-H. **(C)** FSC/CMCS. **(D)** Contents of secondary structures in the silk protein (%).

The polypeptide chain conformation determines the solubility and surface morphology of the fibroin and sericin. Hence, infrared spectroscopy was used to measure the conformation of silk fibroin in the samples (Ling et al., 2011; Wang et al., 2014a; Wang et al., 2014b). All the protein spectra in FSC included the characteristic absorption band of the active groups (near 3,400 cm^−1^) such as hydrogen-bonded amino or carboxyl groups, which were located at the amide I and amide II bands in the range of 1700–1,500 cm^−1^, as determined by the properties of the amino acids comprising the proteins. Among them, the amide I (1700–1,600 cm^−1^) and amide II (1,600–1,500 cm^−1^) bands could be applied to the protein secondary structure (Qiang et al., 2012; Koebley et al., 2015). The spectrogram following the high pressure and temperature treatment of FSC-H without CMCS showed that the absorption peak of the amide II band at 1600–1,500 cm^−1^ had disappeared and that the peak of the amide I band was weakened, indicating that the high temperature may have changed the secondary structure of the protein. In other words, the β-folded structure of the silk protein can be changed through rapid heating, generating a random coil (Cebe et al., 2013). The spectrogram of CMCS with peaks at 1,650 cm^−1^ and 1,420 cm^−1^ corresponded to the stretching vibration peaks of C=O double bonds and C-O single bonds in carboxylic acid, respectively, indicating that the sample contained carboxyl groups. These peaks are important signs that differentiate N and O CMCS from chitosan (Wu et al., 2012). The strong and wide absorption peak observed at 3,408 cm^−1^ was the stretching vibration absorption peak of O-H and N-H. The absorption peak at 1061 cm^−1^ was the stretching vibration absorption peak of C-O generated from the primary alcohol. The absorption peaks at 2,918 cm^−1^ and 1,327 cm^−1^ corresponded to the stretching and bending vibration absorption peaks of C-H bonds, respectively (Figure 3A). The secondary structure was analyzed using the amide I band of the silk protein, which was resolved by deconvolution and Gaussian curve fitting. The amide I bands (1,600–1700 cm^−1^) were selected for quantitative analysis of the secondary structures of the samples (Figure 4). The results showed no other characteristic peaks appeared in the infrared spectra of any of the samples after hot pressing, and no displacement of the characteristic peaks, indicating that hot pressing did not change the basic structure of the FSC. When CMCS was added to the FSC, the trends of increasing random coil content with hot-pressing were distinct, while the α-helix and β-turn contents showed slight irregular fluctuations.

#### 3.2.2 XRD characterization

XRD was used to further reveal the structures of each sample for different treatment times (Figure 3B). The peak value appeared at 2θ = 20.2°, showing that CMCS retained only From II. The broadening of the peak was caused by the amorphous nature of the polymer: the introduction of carboxymethyl destroyed the hydrogen bond of the chitosan molecules. The molecular structure of CMCS has more amorphous substances, which significantly reduced its intensity and moved to a wider diffraction angle; thus, the continuous peaks appeared at 2θ = 10–40° (Samuels, 1981; Yin et al., 2017). FSC had a strong silk II characteristic diffraction peak at about 20° and a weak silk I characteristic diffraction peak at about 29°. A wider diffraction peak was observed at about 20°, which was attributed to the β-sheet structure of FSC (Ming et al., 2017; Yu et al., 2017). The wide diffraction peak and high diffraction baseline suggested the presence of amorphous fibrin in FSC. The FCS/CMCS composite material showed a significantly decreased cocoon crystallinity and a shifted peak value at 2θ = 29° to the right. Thus, CMCS further changed the crystal structure of FSC, leading to the complete transformation from a crystalline to an amorphous region. In addition, the amorphous structure without obvious signal at 2θ = 41° might have resulted from the cross-linking of FSC/CMCS, and the change of the FSC crystal by polymer nanofibers formed under high temperature. The XRD of FSC-H and the FSC/CMCS composite material were similar. Under high temperatures and pressures, degumming of the silkworm cocoon occurs, and the sericin content changes, further altering the crystallinity of the FSC. The high pressure mainly decreased the characteristic diffraction peak (α-helix) of silk I and the characteristic diffraction peak (β-sheets) of silk II. Similarly, for the FSC/CMCS composite material, high temperature and high pressure mainly decreased the characteristic diffraction peak (β-sheets) of silk II while retaining the characteristic diffraction peak of silk I (α-helix), consistent with the FT-IR results.

#### 3.2.3 Thermogravimetric characterization

Thermogravimetric analysis was used to evaluate the thermal decomposition curve of the samples (Figure 3C). The heat–weight curve showed an initial weight loss at approximately 100°C due to water evaporation (Wang et al., 2017). When the temperature was increased to 210°C, the FSC began to lose weight, indicating the beginning of the thermal decomposition reaction. The weight loss rate reached a maximum at 318°C. The thermogravimetric curve of FSC-H showed a significant thermogravimetric phase with increasing temperature, with a weightlessness temperature of 329°C. The thermogravimetric stability of FSC-H was higher than that of the cocoon at room temperature, likely because the high temperature softened the sericin protein and enhanced the bonding between the fibers and layers. Hot compression can also promote decreased moisture content in cocoons (Wang et al., 2018b). Two thermogravimetric processes are reflected in the thermogravimetric curve of FSC/CMCS. The heat loss near 130°C was the evaporation of water molecules in the composite material. With increasing temperature, the material showed a significant thermogravimetric phase; i.e., the thermogravimetric phase of FSC. The peak temperature of the weight loss decomposition was 294°C (Figure 3D), and the thermal weightlessness at this stage was the local breaking of the cocoon molecular chain and the breaking of intermolecular forces. The thermogravimetric temperature of the CMCS material was 280°C, which corresponded to the dehydration of sugar rings in the CMCS molecules and the decomposition and breaking of carboxymethyl in the polymer. This temperature was lower than that for the thermal decomposition of chitosan (Song et al., 2006).

### 3.3 Tensile properties

**FIGURE 5:**
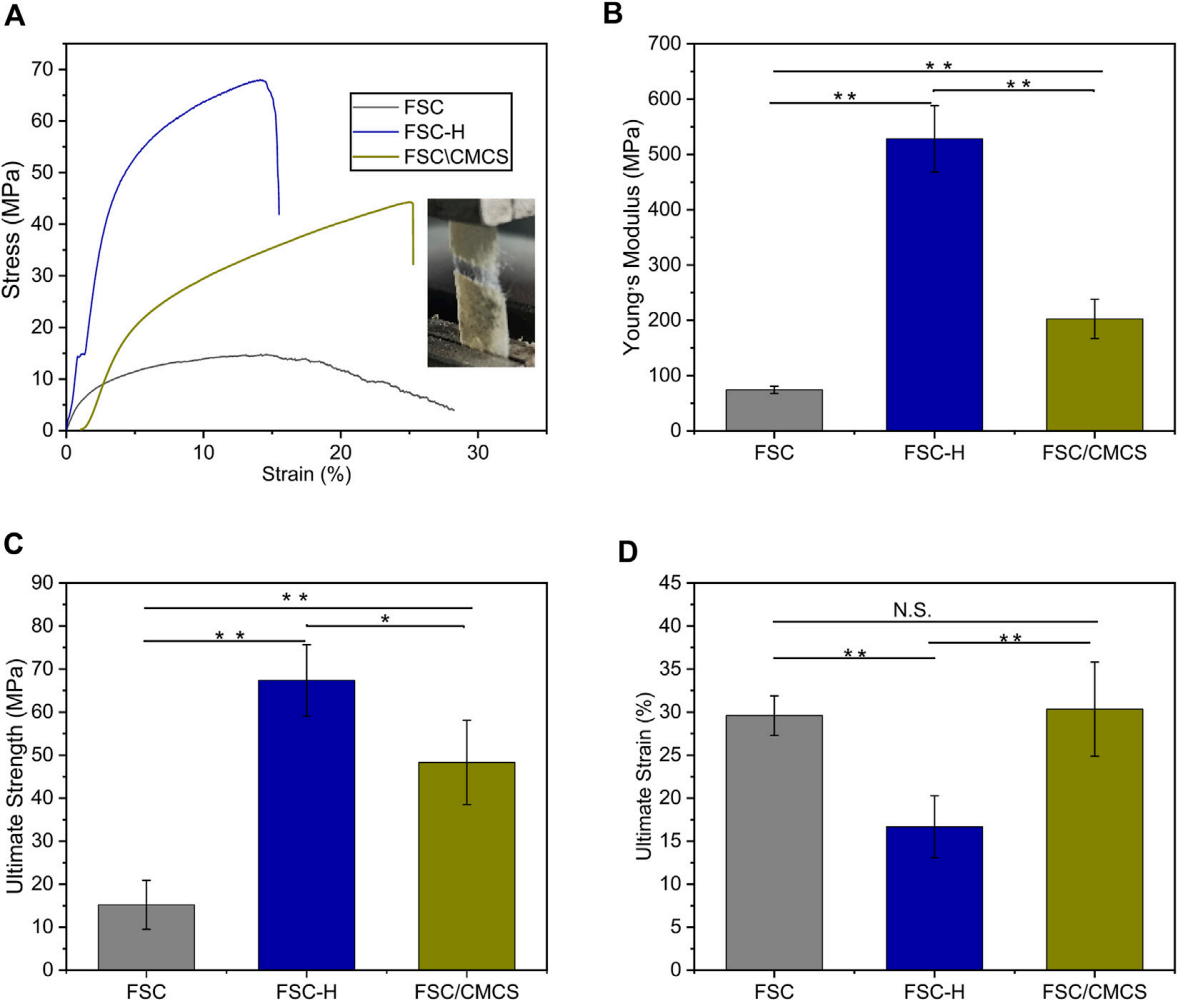
Stress–strain curves and images. **(A)** Stress–strain curves and images of the setup used to stretch the samples and measure their tensile mechanical properties. **(B)** Young’s modulus histograms. **(C)** Ultimate strength histograms. **(D)** Ultimate strain histograms. N.S.: no significant difference. (*p < 0.05, **p < 0.01). The error bar: mean ± SEM (*n* = 3).

Figure 5 shows representative tensile strength–strain curves for FSC, FSC-H, and FSC/CMCS. In this study, FSC alone showed excellent mechanical properties. The high temperature and high pressure significantly affected the mechanical properties of the samples. The hot-pressed sample curve showed a slight fluctuation when the low elongation was 2–3% (Figure 5A), which was attributed to the compact structure of the compression composite material. Figure 5B shows the Young’s modulus values. FSC had a relatively low Young’s modulus (74.25 ± 6.68 MPa). After thermal processing, the Young modulus and tensile strength both increased, with Young’s modulus values for FSC-H and FSC/CMCS of 528.27 ± 60.32 MPa and 202.55 ± 35.34 MPa, respectively. The maximum stresses were 67.36 ± 8.30 MPa and 48.29 ± 9.77 MPa, respectively. The final strain of FSC/CMCS was 30.34% ± 5.47 and the high temperature and pressure improved the stress limit of the FSC-H samples (Figures 5B,C). The tensile strength increased linearly and sharply at low strain. The yield was obvious: it increased continuously with strain until the stress reached the maximum value and then decreased rapidly. This indicated that under high pressure, the cocoon became strong but brittle, as the strain decreased to 16.67% ± 3.59 (Figure 5D). FSC had higher fiber volume fraction and bonding density under hot pressing; the pure fused sericin-binding matrix also made the cocoon brittle.

### 3.4 Biocompatibility of FSC/CMCS

**FIGURE 6:**
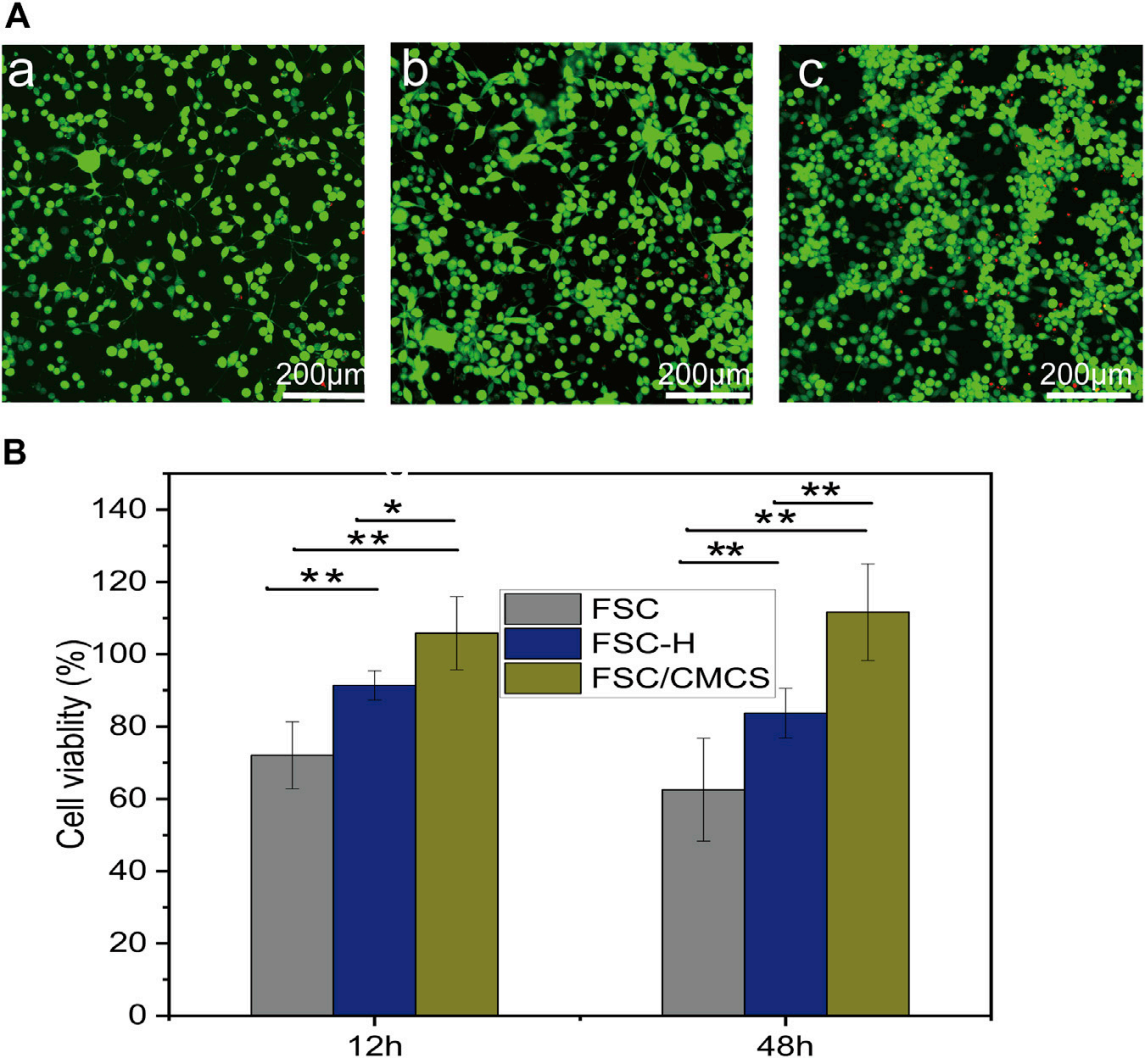
Cytotoxicity assays of L929 murine fibroblast cells upon treatment with basic DMEM. **(A)** Growth of L929 cells treated on **(a)** FSC, **(b)** FSC-H, and **(c)** FSC/CMCS membranes. **(B)** Cytotoxicity of L929 cells after 12 and 48 h of contact with leaching liquors obtained from the FSC, FSC-H, and FSC/CMCS membranes. Live cells stained with Calcein-AM dye produced an intense uniform green fluorescence. Dead cells stained with EthD-I dye emitted bright red fluorescence. Error bar: mean SEM. (*n* = 3, *p < 0.05, **p < 0.01).

The standard MTT method was used to evaluate the cytotoxicity of FSC, FSC-H, and FSC/CMCS samples on mouse L929 fibrocytes. Various leaching solutions were extracted from the samples to treat the cells. The resulting cell viabilities are shown in Figure 6A. The results revealed no cytotoxicity in samples regardless of the incubation time. The FSC-H and FSC/CMCS samples showed high biocompatibility, and the cell viability exceeded 90%. The metabolic activity of cells in direct contact with the sample extract was higher than that in cells in contact with the control extract. In addition, the cells treated with all extracts emitted green fluorescence signals, with few dead cells showing red fluorescence. The preliminary evaluation of the biocompatibility of FSC/CMCS materials showed that these materials had no cytotoxicity. Regarding the FSC/CMCS membrane, the appropriate CMCS content improved the biocompatibility of the materials. These results indicated that compared to other materials, the cells in the FSC/CMCS leaching solution showed significantly improved viability. After 12 and 48 h, the cell viability was not significantly improved for FSC. However, the cells appeared to be more evenly distributed and organized into clusters in the samples exposed to FSC/CMCS (Figure 6B).

### 3.5 Effects on wound healing

Based on the sound performance of the FSC/CMCS mixed membrane, we further studied its effect on wound healing as a dressing in rats. At present, the full-thickness skin wound model is generally adopted to evaluate the wound-healing effects of different types of wound dressings.

**FIGURE 7:**
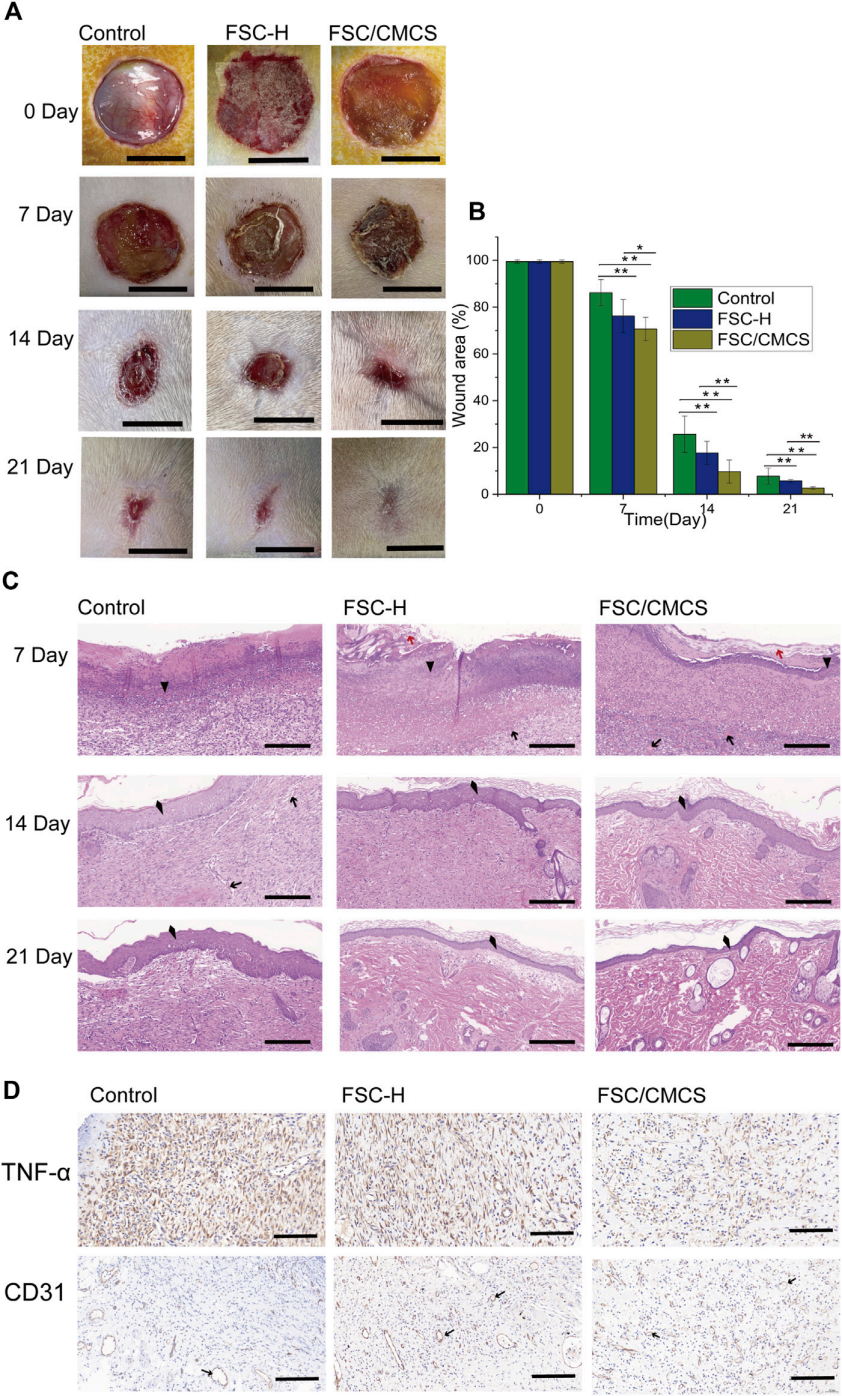
Digital images of macroscopic wound conditions and histological images. **(A)** Digital photographs of wounds taken on days 7, 14, and 21. **(B)** Percentages of wound size compared to the initial sizes. **(C)** H&E staining of sections of wounds treated with different materials on different days, respectively. **(D)** Immunostaining of TNF-α and CD31 in granulation tissues after 7 days. In immunohistochemistry, the nucleus is blue and the positive expression is brownish yellow. Arrows, red arrows, triangles, and quads represent new blood vessels, silk fiber, inflammatory cells, and new epithelium, respectively. Scale bars in **(A)** are 1 cm, and in **(C)** and **(D)** are 100 μm, vs. the control group, (*n* = 3, *p < 0.05, **p < 0.01).

During the repair period, the wound size of the control group was the largest and the wound areas treated with wound dressings significantly decreased with improved efficiency. After 7 days, the damaged skin contact interface materials covered by the FSC-H film were partially crimped, while the wound surface treated by FSC/CMCS mixed film was closely fitted. The wound area of the control group was 86.21 ± 5.64% (Figure 7A). Under the same conditions, the FSC-H and FSC/CMCS dressings effectively reduced the wound sizes and inflammation. After 7 days, the wound areas were 76.21 ± 7.07% and 70.71 ± 4.95%, respectively. No obvious infection was observed between the implanted material or the surrounding tissue, including pyogenic cavity formation and tissue necrosis, as well as no significant swelling or inflammatory reaction. After FSC/CMCS dressing for 14 days, the wound size and inflammatory reaction were effectively reduced, with a wound area of 9.71 ± 4.94%. After FSC/CMCS dressing for 21 days, the wound had almost healed (Figure 7A), with a wound area of 2.66 ± 0.78% (Figure 7B). In comparison, the wounds of the control and FSC-H groups retained small masses and showed slow healing.

Wound healing is a special biological process associated with tissue growth and regeneration. This process can be divided into four stages: hemostasis, inflammation, proliferation, and maturation (Shevchenko et al., 2009). The large number of inflammatory cells in the present study indicated that they were mainly distributed on the edges of the wounds and the biomaterials that fell off at 7 days (Figure 7C). The histological analysis revealed a trend of a higher number of microvessels in the FSC/CMCS group compared to those in the other groups. The comparative study of various materials revealed that the FSC/CMCS composite membrane significantly promoted microvessel production in granulation tissue at the wound healing site. After 14 days, simple epithelium was observed on the granulation tissue in each group due to basal cell proliferation at the edges of the wounds and migration to the center of the wound. Meanwhile, the number of microvessels in each group began to decrease. In the FSC/CMCS group, the number of fibroblasts gradually decreased; visible epidermal tissue was regenerated; and a small number of arterioles, venules, and hair follicle cells appeared, indicating that gradual growth and maturation of the granulation tissue. After 21 days, the granulation tissue began to transform into fibrous connective tissue containing spindle fibroblasts, dense collagen, elastic fibers, and other extracellular components. Among the membrane samples, FSC/CMCS showed the best recovery effect, which was closest to normal skin tissue based on the characteristics of the tissue slices. Immunohistochemical analysis of the secretion of CD31 and tumor necrosis factor (TNF-α) on the wound bed, the production of material neovascularization, and the effectiveness of infection prevention (Pham et al., 2008; Basu et al., 2009; Zhang et al., 2022) (Figure 7D) revealed a large amount of CD31 and a small amount of TNF-α in the FSC/CMCS group after 1 week of healing, indicating that the composite material effectively inhibited inflammatory reactions and promoted angiogenesis.

## 4 Discussion

The skin is the largest organ in the body and performs many important functions. Recently, increased research in the medical and biomaterial fields has focused on the development of new scaffold materials for skin tissue engineering to rapidly heal skin lesions and effectively repair the tissue functions of skin defects, especially skin defects that are difficult to cure.

The material properties can be optimized by mixing and other special methods, thus taking full advantage of both materials. FSC are a multi-layer material composed of continuous silk fibroin fibers reinforced with sericin. Silk fiber is widely used as a reinforcing material due to its low density, outstanding mechanical properties, and sound sustainability (Chen et al., 2001; Zhao and Asakura, 2001). In the present study, sericin fiber was not clearly observed on the FSC-H and FSC/CMCM surface, indicating that sericin was easy to soften and overflow at 130°C and 20 Mpa, and was fused on the silk fiber. Meanwhile, compression at high temperatures activated the “heat reflux” of sericin. During this process, the material undergoes deformation, matrix flow, and curing of the composite structure (Alessio et al., 2019). Under hot pressing, the sericin-binding matrix showed thermoplastic deformation. The inter-fiber and interlayer bonding increased due to the integral sericin binder after hot pressing. Furthermore, CMCS was integrated into the gaps on the FSC/CSCM membrane; thus, gaps and single fibers were not observed on the cross-sections and the structure was more compact. The CMCS fused with the dissolved sericin, with a refractive index similar to that of silk fiber (Xu et al., 2020). The smooth surface of the material and its matching refractive index both ensured the high transparency of the FSC/CMCS membrane (Figure 2F).

**FIGURE 8:**
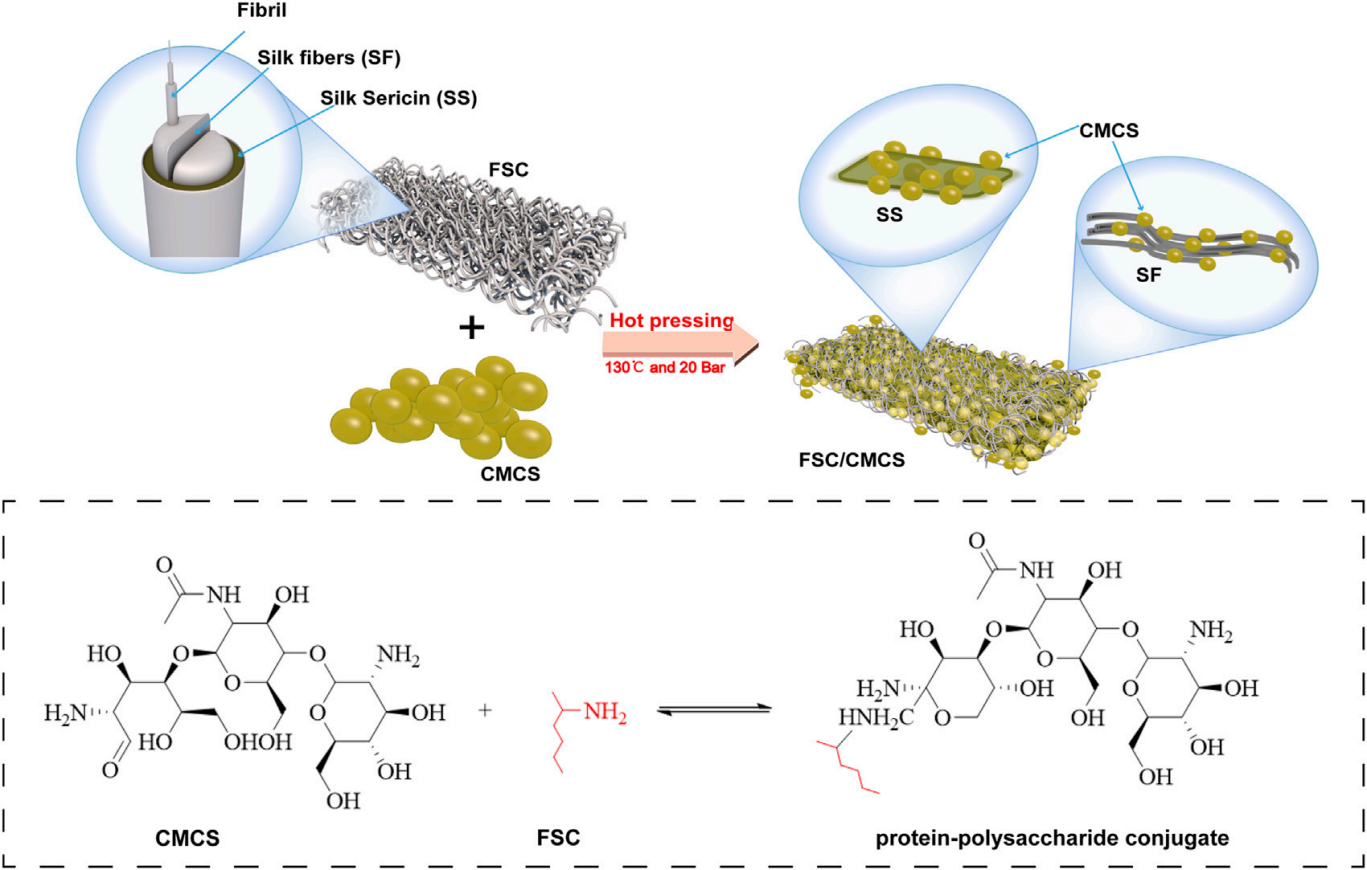
schematic mechanism interpretation of the FSC/CMCS membrane, protein–polysaccharide conjugates are formed by hot pressing to generate a Maillard reaction.

The protein secondary structures were determined by deconvolution of the curve-fitting of the amide I band. The quantitative analysis of the secondary structures of the samples showed infrared spectra and no other characteristic peaks, indicating that hot pressure did not change the basic structure of the FSC (Figure 4). The main difference between FSC and FSC/CMCS on the infrared spectrum was the amino and amide II band. FSC had an obvious amide II band with an absorption peak at 1,524 cm^−1^, while the absorption peak of the amide II band was not obvious in FSC/CMCS. Due to the low content of -NH2, the absorption peak of FSC/CMCS was not obvious at 1,524 cm^−1^ in the infrared spectrum. Comparison of CMCS and FSC/CMCS showed that the absorption peak was not obvious at 1,418 cm^−1^ (carboxyl) and 1,061 cm^−1^ (ether bond, C-O), indicating their extremely low concentrations in FSC/CMCS. Therefore, the FSC/CMCS may undergo a Maillard reaction under high temperature and high pressure to generate a protein–polysaccharide conjugate. In industrial production, this preparation method is simple and environmentally friendly, without requiring the use of enzymes or cross-linking agents. The functional properties of proteins can be further improved by covalent bonding with polysaccharides, which involves a complex network of non-enzymatic reactions resulting from the initial condensation between an available amino group and a carbonyl-containing moiety, usually a reducing sugar (Oliver et al., 2006). The Maillard reaction is a spontaneous and naturally occurring reaction, in contrast to acetylation, deamidation, succinylation, and other chemical methods used to improve the functional properties of proteins. This reaction is greatly accelerated by heat and requires no extraneous chemicals. The increased random coil content in the present study may be related to the Maillard reaction. Hence, this reaction may be the most promising approach to improve the functional properties of proteins. For example, the allergen structure of proteins can be masked by conjugation with polysaccharides (Kato, 2002). After adding CMCS to FSCs in the present study, the thermal weight loss stability of the composite material improved due to the amorphous structure of CMCS. The addition of CMCS increased the interaction between CMCS and the cocoon protein intergroup under high temperatures and pressures to generate a protein–polysaccharide conjugate; this composite material showed a cross-linked molecular structure, with relatively better stability (Figure 8).

Silkworm cocoon is a synthetic nonwoven composite material with a multi-layer structure, in which a certain amount of sericin functions as the matrix binder. The multilayered structure of the silkworm cocoon is the disordered arrangement of silk protein wrapped by sericin. This layered interlaced structure provides the mechanical properties of the silkworm cocoon (Qiang et al., 2012). CMCS filled by hot pressing showed synergistic effects with molten sericin matrix, with sound tensile properties and value in clinical applications. Hence, FSC/CMCS can better enhance wound contact due to its excellent elasticity. In addition, the membrane helped reduce wound tension, prevent excessive wound movement, and ameliorated external forces leading to dressing damage.

Recent studies have reported that the sericin protein and chitosan can simulate cell migration and promote cell viability (Liang et al., 2017). The FSC/CMCS in the present study contained sericin and an appropriate amount of CMCS, which improved the biocompatibility of the materials. Moreover, the FSC/CMCS supported fibroblast adhesion, which aids in the application of wound dressings. The effective wound healing by FSC/CMCS may be attributed to the synergistic effects of the antibacterial effect of sericin and the CMCS complex after cross-linking and the characteristics of cell adhesion and growth promotion. Therefore, FSC/CMCS tested under the same conditions reduced the wound size and inflammation and promoted healing. To sum up, the smallest wound size at the macro level and the complete tissue repair at the micro level supported the optimal healing efficiency of the FSC/CMCS hybrid membrane.

The SEM images of the FSC surface in this study showed that the composite structure was multi-layered, uniform, and highly densified after hot pressing. The FSC/CMCS composite membrane was made by adding CMCS. Under hot pressing, FSC and CMCS produced protein–polysaccharide conjugates through the Maillard reaction, resulting in a high-performance blended membrane with increased unit intensity. This membrane also showed great mechanical properties. The results of the *in vitro* cell experiment demonstrated the cell compatibility of the FSC/CMCS hybrid membrane. The sericin protein of FSC was cross-linked with CMCS through hot pressing, which improved wound healing from both the macro and micro aspects.

## 5 Conclusion

The results of this study demonstrated the practicality of hot pressing FSC and CMCS. During this process, the material undergoes deformation, matrix flow, and curing of the composite structure. Under hot pressing, the sericin-binding matrix showed thermoplastic deformation. From the macro- and micro-aspects, this hybrid membrane improved the efficiency of full-thickness wound repair *in vivo* and accelerated the recovery of whole-cortex wounds. The recovery conditions were further confirmed by the results of the histological and immunohistochemical analyses, which demonstrated the potential application prospect of this material in wound treatment. The results showed that the FSC/CMCS can provide a low-cost and environmentally friendly process for making dressings. Therefore, the FSC/CMCS composite material shows considerable potential in clinical applications, especially for wound healing. However, other relevant indexes require evaluation to elucidate the details of wound healing.

## Data Availability

The raw data supporting the conclusion of this article will be made available by the authors, without undue reservation.
